# Scenes, Spaces, and Memory Traces: What Does the Hippocampus Do?

**DOI:** 10.1177/1073858415600389

**Published:** 2015-08-14

**Authors:** Eleanor A. Maguire, Helene Intraub, Sinéad L. Mullally

**Affiliations:** 1 Wellcome Trust Centre for Neuroimaging, Institute of Neurology, University College London, London, UK; 2 Department of Psychological and Brain Sciences, University of Delaware, Newark, DE, USA; 3 Institute of Neuroscience, Newcastle University, Newcastle, UK

**Keywords:** hippocampus, memory, space, navigation, scenes, imagination, scene construction, boundary extension, perception, future thinking, simulation

## Abstract

The hippocampus is one of the most closely scrutinized brain structures in neuroscience. While traditionally associated with memory and spatial cognition, in more recent years it has also been linked with other functions, including aspects of perception and imagining fictitious and future scenes. Efforts continue apace to understand how the hippocampus plays such an apparently wide-ranging role. Here we consider recent developments in the field and in particular studies of patients with bilateral hippocampal damage. We outline some key findings, how they have subsequently been challenged, and consider how to reconcile the disparities that are at the heart of current lively debates in the hippocampal literature.

## Memory and Space

**Figure 1.**
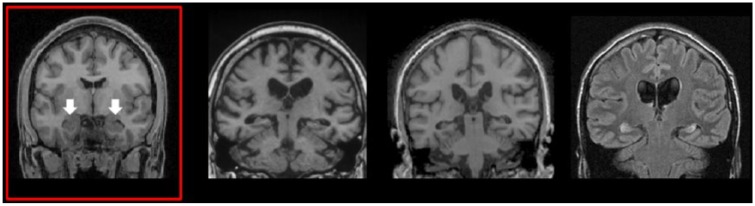
The human hippocampus. Coronal sections from magnetic resonance imaging brain scans: left panel (red box), the arrows indicate the two hippocampi of a healthy control participant. The other panels show scans of patients with bilateral hippocampal damage.

Since the 1950s, the hippocampus, a structure located deep in the temporal lobes, has been one of the most intensively studied regions of the brain (Fig. 1), motivating in excess of 100,000 research articles in the past 60 years. There are two principal reasons why the hippocampus came to provoke such interest. Scoville and Milner (1957) reported the case of patient HM who became profoundly amnesic following removal of his temporal lobes, including the hippocampi, for the control of intractable epilepsy. Consequently, the hippocampus became associated with episodic or autobiographical memory, the memory for our personal past experiences. Just over a decade later, O’Keefe and Dostrovsky (1971) working with rats made the (Nobel-prize winning) discovery that there were neurons in the hippocampus that exhibited location-specific firing—place cells. This suggested a primary role for the hippocampus in representing space.

In the following years, these two lines of inquiry into the hippocampus existed largely independently, with few substantial attempts to integrate findings across domains (Burgess and others 2002; Eichenbaum and Cohen 2014). However, this changed in the early 2000s when data emerged that broadened the remit of the hippocampus even further, which in turn spurred on attempts to understand why the hippocampus seems to play a critical role in a wider range of cognitive functions. Specifically, testing patients with bilateral hippocampal damage (Fig. 1), it was found that they not only had autobiographical memory and spatial navigation deficits, but they were also impaired at perceiving scenes (reviewed in Graham and others 2010) and constructing fictitious and future scenes in their imagination (Hassabis and others 2007b). Of note, the patients described the scenes they attempted to construct as fragmented and lacking spatial coherence.

This scene construction deficit was subsequently replicated in different sets of patients whose hippocampal damage arose from a variety of etiologies (e.g. Andelman and others 2010; Kurczek and others 2015; Mullally and others 2012b; Race and others 2011; Rosenbaum and others 2009). Interestingly, patients were not impaired at perceiving or imagining single objects, the deficits were specific to scenes. Functional magnetic resonance imaging (fMRI) studies in healthy volunteers confirmed the engagement of the hippocampus not only for autobiographical memory (Maguire 2001) and navigation (Spiers and Maguire 2006) but also for imagining fictitious (Hassabis and others 2007a; Zeidman and others 2014) and future scenes and events (Addis and others 2007).

## New Perspectives on Hippocampal Function

In light of these findings a shift occurred in human hippocampal neuroscience and several new theories emerged, which cast the hippocampus in a different light. What they had in common was viewing the hippocampus not as fundamentally mnemonic or spatial, but instead as providing a process that was required not only by memory and navigation but also by imagining the future (Buckner and Carroll 2007; Schacter and Addis 2007). One perspective of particular relevance here is the scene construction theory (SCT; Hassabis and Maguire 2007; Maguire and Mullally 2013). It posits that a primary function of the hippocampus is to facilitate the construction of scenes by allowing details to be martialled, bound, and played out in a coherent spatial context. In this way scene construction is held to be a vital ingredient not only for episodic memory and imagining the future but also for spatial navigation and scene perception (Zeidman and others 2014). Placing scenes at the center of hippocampal information processing has intuitive appeal. For most people, when recalling the past, thinking about the future, and planning how to get somewhere, this typically involves imagining scenes. SCT also makes clear predictions. For example, because autobiographical memory depends on scene construction, the theory implies that it would be impossible for a patient with intact autobiographical memory to have impaired scene construction. Indeed Squire and others (2010) reported that their patients with hippocampal damage but without pervasive autobiographical memory deficits had intact scene construction ability, in line with predictions of SCT.

**Figure 2.**
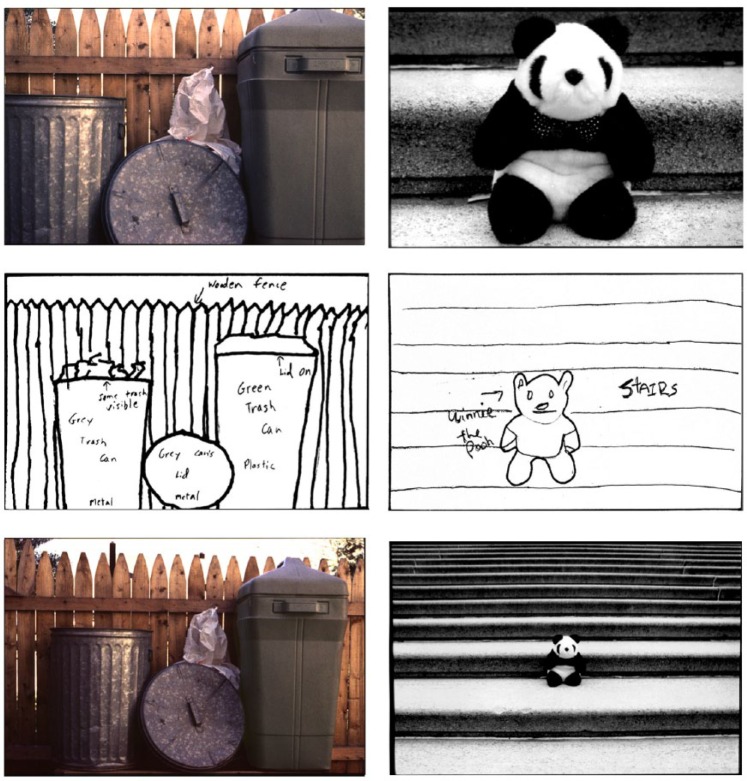
Two examples of boundary extension in drawings. The left column (top) shows a tight close-up of “trash cans by a fence,” which was presented for 15 seconds within a picture sequence. Beneath it is a participant’s drawing from memory, 48 hours later, and below that is a more wide-angled view showing what actually did exist just beyond the picture’s boundaries (based on Intraub and Richardson 1989). The right column (top) shows a tight close-up of “toy bear on steps” that had been presented for 250 ms in a picture sequence. Beneath it is a participant’s drawing from memory minutes later, and below that is a more wide-angled view showing what actually did exist just beyond the picture’s boundaries (based on Intraub and others 1996). Note that drawings from memory included content from beyond the boundaries of the photographs—this anticipatory memory is referred to as boundary extension (BE).

Perhaps the most powerful support for SCT comes from studies of boundary extension (BE; Intraub and Richardson 1989). BE is a ubiquitous cognitive phenomenon where we erroneously remember seeing more of a scene than was present in the sensory input, and occurs because when we view a scene, we implicitly and automatically extrapolate beyond the borders to form an extended internal representation of that scene. In the absence of the original visual input, this extended scene is misremembered instead of the original input, causing a memory error (Fig. 2). Of note, BE only occurs in relation to scenes and not single isolated objects (Gottesman and Intraub 2002). BE also depends on scene construction as this is required in order to represent what might be beyond the view. BE is evident in drawings from memory (as in Fig. 2), recognition memory, and boundary reconstruction tasks—even when subjects are blindfolded and tested haptically (Intraub and others 2015). Crucially, Mullally and others (2012b) found that patients with bilateral hippocampal damage and scene construction deficits had attenuated BE on all of these tasks. This resulted in more accurate memory performance by the patients compared with the control subjects and as such the BE findings cannot be explained by the patients’ memory impairment. Subsequently, Chadwick and others (2013) corroborated these findings using fMRI showing that the initial BE effect, where the view is extended at the point of scene perception, is associated with increased hippocampal activity in healthy subjects.

## Recent Challenges

These BE results provide compelling evidence that a core function of the hippocampus may be scene construction. Nevertheless, since we last reported here (Mullally and Maguire 2013), new data have emerged that appear to contest the idea that the hippocampus is more than a pure memory structure, while also calling into question the BE patient findings that are so central to this view. Here we consider three types of challenge in an effort to understand and reconcile current disparities in the literature.

### Conceptual Issues

Kim and others (2015) maintain the sole function of the hippocampus is mnemonic. Evidence in support of this view comes from their findings of preserved spatial navigation (Teng and Squire 1999), scene construction, imagining the future (Squire and others 2010) and normal BE (Kim and others 2015) in patients with bilateral hippocampal damage. Moreover, they suggest that scene perception deficits are in fact due to the patients’ underlying memory problem (Kim and others 2011). The difficulty with this position is the substantial evidence from numerous different laboratories of impaired spatial navigation (reviewed in Clark and Maguire 2015), scene construction and imagining the future (reviewed in Maguire and Mullally 2013) and deficits in scene perception (reviewed in Graham and others 2010) in patients with lesions to the hippocampus. There is also a wealth of animal work linking the hippocampus to spatial navigation (Andersen and others 2006) and even recently to imagining the future (Ólafsdóttir and others 2015). It is important to reiterate that SCT and the other new perspectives do not deny that the hippocampus has a vital role to play in memory. But the general consensus in the field currently is that memory, space, scene perception, and scene construction are not mutually exclusive, and there is considerable credible evidence that the hippocampus plays a role in all of these cognitive functions.

There is no easy way for the memory-only model to account for these contrary findings, unless we presume that all of them are in some way due to a memory deficit. It is for this reason the BE findings are particularly important. The memory error here is a *constructive* memory error involving scenes. This is different from instances of memory loss, for example, in a recognition test for scenes or details recalled (e.g. Kim and others 2015). However, recent BE research with hippocampal patients has provided a seeming counterpoint to Mullally and others’ (2012b) BE patient study.

**Figure 3.**
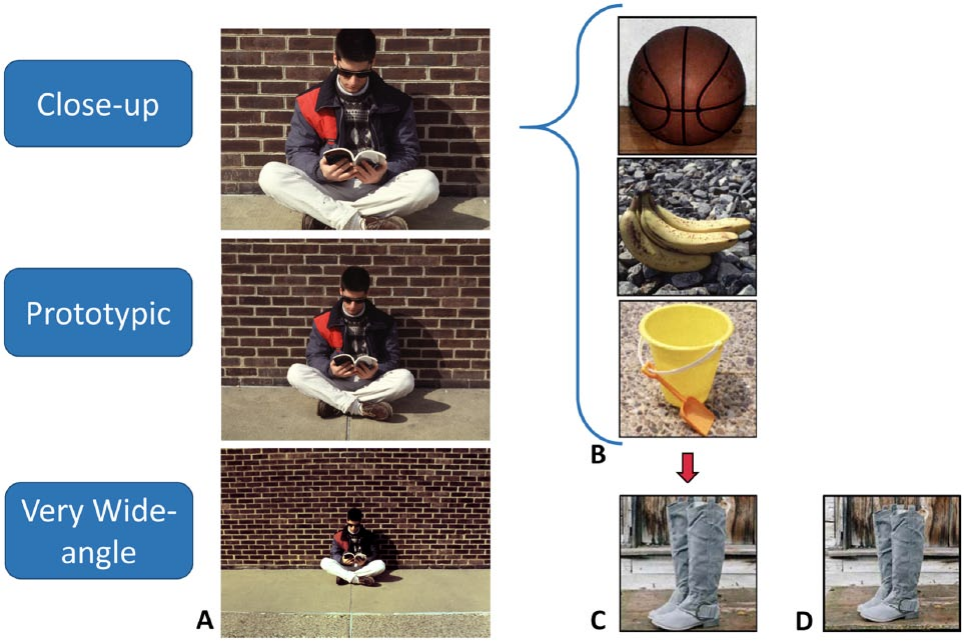
Tight close-ups elicit the greatest boundary extension. (A) A sample range of stimuli for which boundary extension was greatest for the tight close-up, decreased for the prototypic view, and led to no directional boundary error for the very wide-angle view (based on Intraub and others 1992). (B) The three tight close-ups used in the drawing task in Mullally and others (2012b); on average, objects fill 43.4% of the photographs. (D) A photograph used in Kim and others (2015), in which the pair of boots cover about 30% of the photograph (reproduced with permission from Proceedings of the National Academy of Sciences USA). (C) The same photograph with the scope adjusted to create a tighter close-up that falls within the range of Mullally and others’ (2012b) photographs (boots now cover about 39% of the photograph), thus highlighting the change in proximity to the boundaries and amount of background space that differences in scope can make.

Whereas Mullally and others (2012b) found that patients who drew simple scenes (as in Fig. 3B) from memory, exhibited less BE than control participants, Kim and others (2015) reported that when their patients drew scenes such as the one in Figure 3D, no difference in BE was observed compared with control participants. Similarly, in a rapid serial presentation task, when boundary memory for photographs each presented for 250 ms were tested after a 250 ms masked retention interval, patients in Mullally and others (2012b) exhibited less BE than the control participants, whereas in Kim and others (2015), again, no difference was observed. Kim and others argued that contrary to SCT, scene construction must be mediated by brain areas other than the hippocampus (perhaps the parahippocampal cortex). How can we reconcile these seemingly contradictory data? Perhaps the nature of the patients and some differences in the methodologies hold clues.

### Patient Characteristics

The patients studied in Kim and others (2015) were the same as those in the study of Squire and others (2010) where it was reported that these patients did not exhibit the scene construction deficit that characterized Mullally and others’ (2012b) patients. Kim and others (2015) suggested that patients’ lesions in Mullally and others (2012b), were not truly focal to the hippocampus and damage to other areas might have contributed to their impairments. However, using high-resolution MRI scanning and multiple analysis techniques, no other pathology was found in the Mullally patients. Kim and others (2015) also questioned the degree of hippocampal volume loss in Mullally and others’ (2012b) patients. In fact, Kim and others (2015) made a factual error on this point. They incorrectly claimed that two of Mullally and others’ (2012b) patients had hippocampal volume loss greater than 70%. As stated by Mullally and others (2012b), the volumes were reduced to (not by) 68.7% to 78.33% of normal.

Kim and others (2015; see also Squire and others 2010) suggested that pathology arising from limbic encephalitis (LE) is invariably more diffuse compared with aetiologies like anoxia and that this may be an issue where LE patients are included in a cohort (such as in Hassabis and others 2007b and Mullally and others 2012b). However, postmortem studies of certain types of LE document selective hippocampal damage (Dunstan and Winer 2006; Khan and others 2009; Park and others 2007), and even in non-LE patients scene construction deficits have been found. On the other hand, patients who have been described as having selective hippocampal-damage from non-LE pathologies can have wider brain damage (e.g., Kim and others 2015: patient DA, heroin overdose, bilateral globus pallidus lesions; patient KE, toxic shock syndrome, basal ganglia lesions). Arguments about the selectivity of lesions are unhelpful because many pathological processes produce widespread brain damage, but only those rare patients with apparently selective hippocampal lesions are typically included in studies where the prime or sole interest is in the hippocampus (as in Mullally and others 2012b; see Clark and Maguire 2015 for more on this). For these reasons it is difficult to dismiss Mullally and others’ (2012b) results on the basis of their choice of patients.

### Methodological Differences

Because Kim and others’ (2015) patients did not exhibit a scene construction deficit (Squire and others 2010), a difference in BE would not necessarily be anticipated. However, in testing for a difference between patients and control participants, it is important that the stimuli and test procedures be at least as sensitive as in the original study by Mullally and others (2012b). This is because, although BE is very robust and readily observed, the size of the error tends to be highly variable across participants and scene stimuli, making it challenging to find group differences. This is particularly true in a small group of rare patients such as those with selective bilateral hippocampal damage where the power to detect such differences is low. To enhance the possibility of detecting a group difference if one is present, it is important to use stimuli that will yield the largest effect and procedures that minimize the impact of other memory distortions (e.g., normalization to the average view; Intraub and others 1992) from interacting with and minimizing BE.

One of the hallmarks of BE, reported first in Intraub and Richardson (1989) and evaluated more closely in Intraub and others (1992), is that BE is greatest for tight close-ups and decreases as more surrounding space is made visible (as in more wide-angle views) until with very wide-angle views, no BE is observed (see Fig. 3A). In subsequent research, drawing from memory, recognition memory, and boundary reconstruction tasks have replicated this hallmark (Hubbard and others 2010; Intraub 2002). Regrettably, Kim and others (2015) did not request any scene stimuli from Mullally and others (2012b) and instead created their own stimulus sets. For the drawing task, whereas Mullally and others (2012b) presented very tight close-ups in which objects filled, on average, 43.4% of the view, minimizing background area as much as they could, Kim and others (2015) presented more wide-angled views in which objects, on average, filled only 30.2% of the photograph. The difference in stimuli between the two studies is apparent in Figure 3. The greater amount of visible background, the smaller the boundary error will be, compressing the possible range of errors, making it more difficult to detect potential differences between groups.

There are other aspects of design that differed between the two drawing studies. The goal in Mullally and others (2012b) was to minimize the number of trials to avoid interference across trials and fatigue. They presented three trials, thereby keeping the number lower than the four trials presented in other studies that used the identical task (Candel and others 2004; Seamon and others 2002). Kim and others (2015) more than doubled the amount, presenting 10 trials. A critical instruction in the drawing task is to tell participants to consider the boundaries of the test window to be identical to the boundaries of the photograph, and to draw the remembered image accordingly. This takes time and concentration, requiring about 2 to 3 minutes to make each drawing (Gottesman and Intraub 1999). By comparison, Kim and others (2015) report that their entire drawing task (presentation and drawing of 10 pictures) required only 11 minutes (rather than 20-30 minutes). The brevity of their drawing task invites questions about possible fatigue with the task or perhaps differences in instructions.

Finally, in the rapid serial presentation task, Kim and others (2015) noted that the disparity between the two studies seemed to rest on differences in the control groups’ performance. Of their own data they reported, “. . . but the effect was weaker than reported previously for control participants . . .” (p. 4772). They suggested that perhaps by chance the participants in their and Mullally and others’ (2012b) control groups might have used different criteria when rating the test picture as showing the same view or a closer or more distant view. This cannot be determined without future research. However, once again, their stimuli were not as tight close-ups as in Mullally and others (2012b). Given a difference between groups, this would be expected to disproportionately affect control participants’ responses, limiting BE. This is especially the case in a rapid serial task such as this when memory is tested only a fraction of a second after stimulus presentation (Chadwick and others 2013; Intraub and Dickinson 2008). For future BE patient research, in light of these considerations, we suggest using stimulus sets that only include very tight close-ups, and objects that fill the space in both the length and width (see Dickinson and LaCombe 2014) to allow the best test of differences in BE. Thus, although on the face of it the data from Mullally and others (2012b) and Kim and others (2015) appear to conflict, disparities between the studies may reflect less about hippocampal function than they do about other factors affecting BE.

## Future Directions

Notwithstanding the clear methodological differences in relation to BE patient studies, it is nevertheless the case that there are reports in the literature of apparently preserved scene construction ability following bilateral hippocampal damage that is sustained in adulthood. As already noted, Squire and others (2010) reported that their patients were not impaired at scene construction or imagining the future. However, their patients did not exhibit pervasive autobiographical memory loss (Maguire and Hassabis 2011). As autobiographical memory depends on intact scene construction it is not surprising that when scene construction was tested it was found to be preserved. These same patients also showed normal BE in Kim and others (2015). Again, with their scene construction intact, one would expect normal BE. Likewise, these same patients were unimpaired on tests of scene perception (Kim and others 2011). This particular set of patients stands in contrast to those tested in numerous other laboratories who have truly dense amnesia for autobiographical events often stretching back a lifetime, and also scene construction and future-thinking deficits (reviewed in Maguire and Mullally 2013). That these latter deficits have been replicated by different research groups suggests they have validity and so the question then becomes why is it that some patients with bilateral hippocampal damage seem unimpaired on the tests of interest.

**Figure 4.**
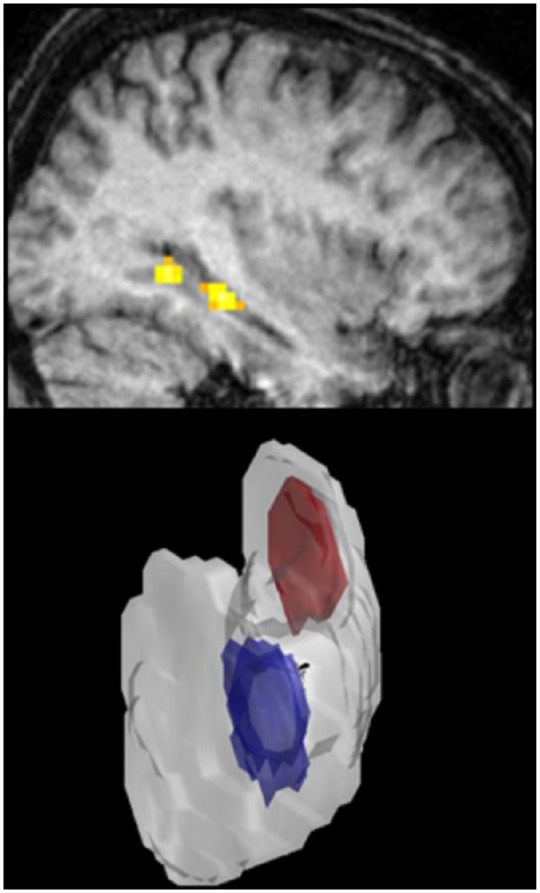
Intrahippocampus considerations. Top panel shows activation of the residual tissue in the right hippocampus of a patient with bilateral hippocampal damage when he constructed scenes in his imagination (from Mullally and others 2012a). Lower panel shows a three-dimensional rendering of a healthy hippocampus from an example participant in the functional magnetic resonance imaging study of Bonnici and others (2012). The blue area indicates where information about recent memories was detectable (anterior hippocampus) and the red area shows where more information was detectable about remote memories (posterior hippocampus).

One patient (P01) in the cohort studied by Hassabis and others (2007b) also showed preserved scene construction ability. When scanned using fMRI while constructing scenes the remnant tissue of his right hippocampus was found to activate (Mullally and others 2012a; Fig. 4). This suggests that for some patients the residual hippocampal tissue may retain some functionality, while in others it may not, thus potentially giving rise to discrepancies in the literature. Going forward, we suggest that more fMRI scanning of patients with apparently preserved functions, particularly when this is unexpected given other results in the field, could illuminate the functionality of residual hippocampal tissue.

The location of damage within the hippocampus may also be important. For instance, Bonnici and others (2012) found that it was possible to decode representations or traces of recent (2-week-old) and remote (10-year-old) autobiographical memories from patterns of fMRI activity in the anterior hippocampus of healthy subjects, while posterior hippocampus contained more decodable information about the remote memories (Fig. 4). This shows that there are functional distinctions within the hippocampus itself that may interact with the location and severity of damage thus leading to different functional outcomes in different patients. The ever-improving resolution of MRI scanning, which is now able to provide information at the hippocampal subfield level, will hopefully start to yield more detailed characterization of intrahippocampal damage which could also inform function.

To conclude, there are heated debates in the field of hippocampal neuroscience in particular surrounding findings in patients with bilateral lesions. On balance, the consensus currently seems to be that the hippocampus may provide computations that are vital for functions such as autobiographical memory, spatial navigation, some aspects of perception and imagining the future. This could involve the construction of spatially coherent scenes that allow for the vivid re-experiencing of memory representations. Science thrives on questioning and critiquing, and it is vital to understand the range of variables that can affect outcomes. Better consideration of methodological details as well as patient characteristics could help minimize or even resolve differences across studies in our quest to understand the hippocampus, which seems so central to our everyday mental experience.
